# Chrysin alleviates imiquimod-induced psoriasis-like skin inflammation and reduces the release of CCL20 and antimicrobial peptides

**DOI:** 10.1038/s41598-020-60050-1

**Published:** 2020-02-19

**Authors:** Hsin-Ju Li, Nan-Lin Wu, Chi-Ming Pu, Chien-Yu Hsiao, Der-Chen Chang, Chi-Feng Hung

**Affiliations:** 1 School of Medicine, Fu Jen Catholic University, New Taipei City, 24205 Taiwan; 2 Department of Medicine, Mackay Medical College, New Taipei City, 25245 Taiwan; 3 Department of Dermatology, Mackay Memorial Hospital, Taipei, 10449 Taiwan; 4 Mackay Junior College of Medicine, Nursing, and Management, New Taipei City, 25245 Taiwan; 5 Division of Plastic Surgery, Department of Surgery, Cathay General Hospital, Taipei, 10630 Taiwan; 6 Department of Nutrition and Health Sciences, Research Center for Food and Cosmetic Safety, and Research Center for Chinese Herbal Medicine, College of Human Ecology, Chang Gung University of Science and Technology, Taoyuan, 33303 Taiwan; 7 Aesthetic Medical Center, Department of Dermatology, Chang Gung Memorial Hospital, Taoyuan, 33305 Taiwan; 8 Department of Mathematics and Statistics and Department of Computer Science, Georgetown University, Washington, DC, 20057 USA; 9 Ph.D. Program in Pharmaceutical Biotechnology, Fu Jen University, New Taipei City, 24205 Taiwan; 10 MS Program in Transdisciplinary Long Term Care, Fu-Jen Catholic University, New Taipei City, 24205 Taiwan; 11 Department of Fragrance and Cosmetic Science, Kaohsiung Medical University, Kaohsiung, 80708 Taiwan

**Keywords:** Interleukins, Skin diseases

## Abstract

Psoriasis is a common non-contagious chronic inflammatory skin lesion, with frequent recurrence. It mainly occurs due to aberrant regulation of the immune system leading to abnormal proliferation of skin cells. However, the pathogenic mechanisms of psoriasis are not fully understood. Although most of the current therapies are mostly efficient, the side effects can result in therapy stop, which makes the effectiveness of treatment strategies limited. Therefore, it is urgent and necessary to develop novel therapeutics. Here, we investigated the efficacy of chrysin, a plant flavonoid, which we previously reported to possess strong antioxidant and anti-inflammatory effects, against psoriasis-like inflammation. Our results revealed that chrysin significantly attenuated imiquimod-induced psoriasis-like skin lesions in mice, and improved imiquimod-induced disruption of skin barrier. Moreover, the TNF-α, IL-17A, and IL-22-induced phosphorylation of MAPK and JAK-STAT pathways, and activation of the NF-κB pathway were also attenuated by chrysin pretreatment of epidermal keratinocytes. Most importantly, chrysin reduced TNF-α-, IL-17A-, and IL-22-induced CCL20 and antimicrobial peptide release from epidermal keratinocytes. Thus, our findings indicate that chrysin may have therapeutic potential against inflammatory skin diseases. Our study provides a basis for further investigating chrysin as a novel pharmacologic agent and contributes to the academic advancement in the field of Chinese herbal medicine.

## Introduction

Psoriasis is a frequently recurring non-contagious chronic skin disease, with obvious manifestations and easy clinical diagnosis^1,2^. The characteristics of this inflammatory disease involve aberrant keratinocyte proliferation, dermal angiogenesis, dendritic cell activation, release of pro-inflammatory cytokines, and recruitment of T lymphocytes, neutrophils, monocytes, and macrophages to skin^3–6^. Typical cutaneous manifestations of erythema and scaling represent vascular and epidermal violation, respectively, and some patients even exhibit psoriasis arthritis, resulting in swelling and inflammation of the joints, and nail lesions, caused by nail deformation^7,8^. The pathogenesis of psoriasis is not yet clear; however, increasing evidence shows that psoriasis is an immune-mediated disease^9,10^, which is related to immune dysfunction, and there is no universally effective therapeutic strategy. In addition, psoriasis can also be associated with cardiovascular disease and other comorbidities, such as hypertension, obesity, dyslipidaemia, metabolic syndrome, diabetes, smoking, and other cardiovascular risk factors^11–13^. According to the previous research and clinical experience, several cytokines are released from immune and inflammatory cells during psoriasis, such as TNF-α, IL-17A, and IL-22^6,14–17^, which are speculated to act directly on human keratinocytes and cause a variety of pathological symptoms.

**Figure 1:**
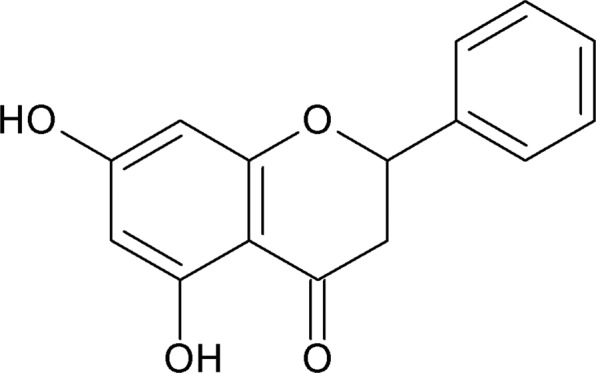
The structure of chrysin.

Polyphenols are natural compounds widely present in the Plantae kingdom^18^. Polyphenols act as protective agents in plants against a plethora of factors, such as ultraviolet radiation and bacterial and pest invasion. Recent studies have shown that polyphenols exhibit several beneficial biological activities, such as anti-angiogenic profile^19,20^, anti-inflammatory, antioxidant, antiviral, anti-allergic, and antitumor^21–25^. As a potent antioxidant reagent, polyphenols can scavenge free radicals in the body, inhibit a series of oxidation reactions to avoid DNA and cell oxidation, and further prevent cardiovascular diseases^26,27^. Flavonoids, also known as bioflavonoids, are the most abundant polyphenols found in the human diet. They are widely found in vegetables, cereals, fruits, rhizomes, flowers, bark, tea, and red wines^28–30^. So far, more than 5,000 different flavonoids have been identified^31^. Previous reports have demonstrated that flavonoids exhibit multiple biological activities. We have found in our previous research that many flavonoids have the effects of anti-photoaging on the skin^32,33^. In addition, in our previous related studies, it has been shown that the treatment of isoflavone extracts is beneficial to improve the inflammation response associated with psoriasis^34^. Isoflavone extracts have ameliorated imiquimod-induced psoriasis-like skin inflammation. It also inhibited the signal transduction induced by TNF-α, IL-17A, and IL-22 in human primary keratinocytes, both in the mRNA and the protein level^34^. Chrysin (C_15_H_10_O_4_) (Fig. 1) is a kind of flavonoid extracted from several plants, such as Passion-flower, honey, and propolis. It exhibits antioxidant^35^, antibacterial^36^, anti-anxiety^37^, hypoglycaemic^38^, antiviral^39^, anti-inflammatory^40^, antitumor^41^ and other activities. In addition to the effects of immunoregulation, our previous studies have also demonstrated that chrysin has photoprotective effects against both UVA- and UVB-induced damage and oxidative stress in HaCaT cells. Furthermore, *in vivo* experiments indicated that topical application of chrysin shows efficient percutaneous absorption and no skin irritation^33^. The advantage of chrysin is not only its antioxidant and anti-inflammatory effects, it is also cheap and can be easily extracted. Therefore, in this study, we will continue to use previous experimental models to evaluate the effects of chrysin on the skin injury and physiological parameters of mice in the imiquimod-induced psoriasis rodent model. In addition, we also use human primary keratinocytes to explore whether chrysin has the effects against the inflammatory response caused by pathogenic cytokines, including TNF-α, IL-17A, and IL-22.

There are many strategies to treat psoriasis; however, the current management is usually unsatisfactory due to the possibility of poor efficacy, severe side effects, high expenses, or frequent recurrence. Therefore, it is urgent and important to develop novel drugs for the treatment of this inflammatory skin disease. In this study, we investigated whether chrysin has the potential to inhibit these cytokine-induced downstream signalling cascades in psoriasis and ameliorate physiological processes in human keratinocytes.

## Results

### Chrysin pretreatment improves skin inflammation and epidermal hyperplasia in imiquimod (IMQ)-induced psoriasis-like model

**Figure 2:**
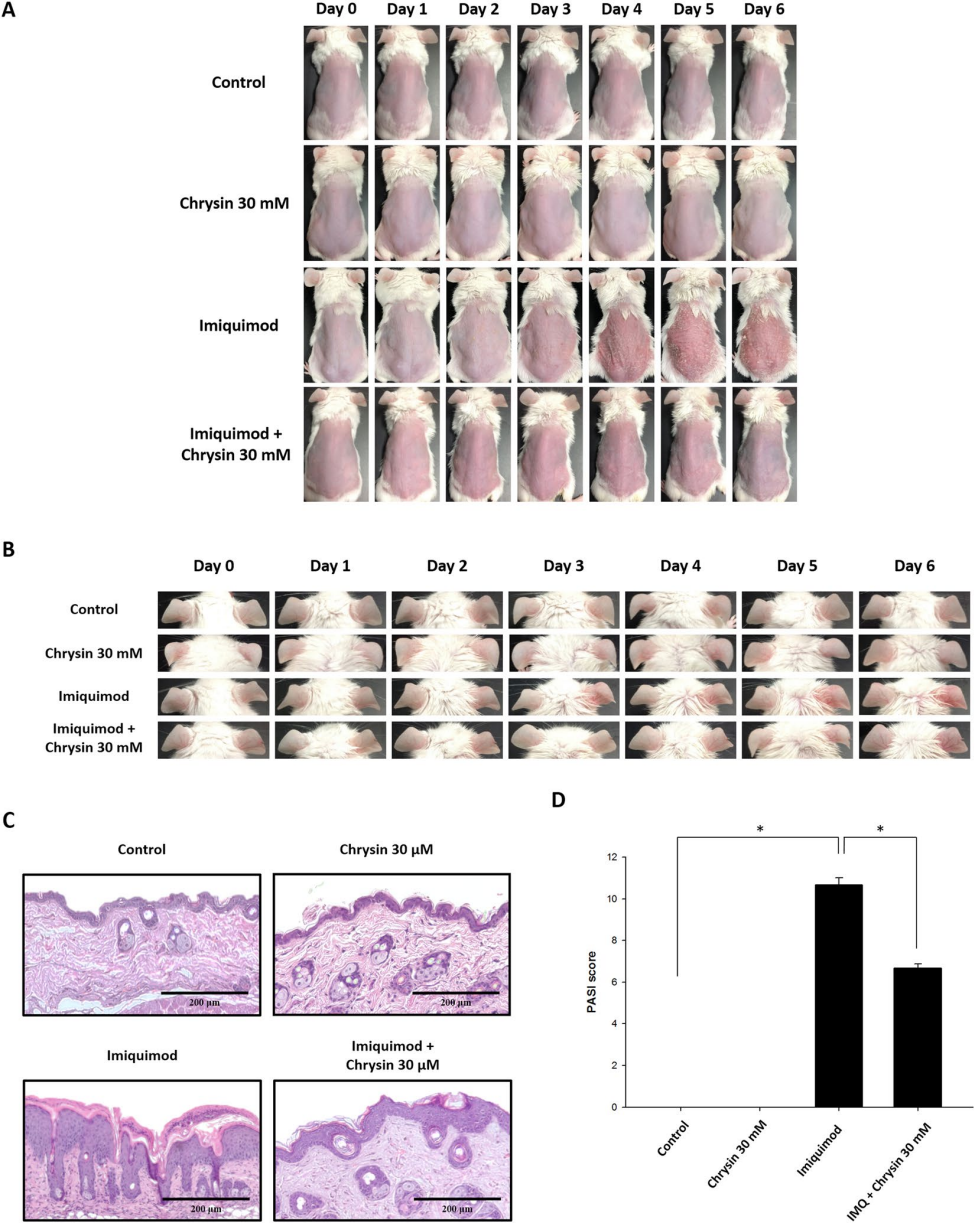
Chrysin improves imiquimod (IMQ)-induced skin inflammation. Chrysin was topically applied for 1 h on the skin and ears of mice before IMQ stimulation or vehicle cream administration for six consecutive days. (**A**) Phenotypic change in the appearance of skin of mice for six consecutive days. (**B**) Macroscopic changes in mouse ears. (**C**) Histological sections of mouse skin stained with haematoxylin and eosin. (**D**) Quantification of mouse skin histology examined using the PASI score. In each group, mice were stimulated with or without IMQ after pretreatment with chrysin. We performed *in vivo* experiments in at least six mice per treatment group, and data represent the mean ± SEM from at least six independent experiments. *p < 0.05 was considered to be statistically significant.

We first explored the anti-psoriatic activity of chrysin in the murine IMQ-induced psoriasis-like skin inflammation model. We compared the macroscopic and physiological characteristics of the control, IMQ-induced psoriasis-like skin, and IMQ-induced psoriasis-like skin with chrysin pretreatment. The results showed that the dorsal skin of the IMQ-treated mice exhibited redness and scaling from Day 4, and later, mouse skin conditions worsened. Similarly, we found that the mouse ears exhibited redness, thickness, and swelling, showing that IMQ induces psoriasis-like inflammation. However, in the group pretreated with chrysin, redness, scaling, swelling, and thickening of the skin and ears of mice were attenuated significantly. These results indicated an inhibitory effect of chrysin on IMQ-induced psoriasis-like skin inflammation (Figs. 2A,B).

The histological effects of chrysin after IMQ-induced psoriasis-like skin inflammation were examined by haematoxylin and eosin (H&E) staining, as shown in Fig. 2C. The histology of IMQ-induced skin displayed significant inflammatory cell infiltrate and hyperkeratosis in the epidermal layer. The skin inflammation induced by IMQ treatment was improved by chrysin pretreatment. The histology of chrysin-pretreated skin exhibited significant improvement with respect to inflammatory symptoms. The total scoring of Psoriasis Area and Severity Index (PASI) showed that chrysin-pretreated groups showed significantly reduced PASI score compared with that of the IMQ group (Fig. 2D).

**Figure 3:**
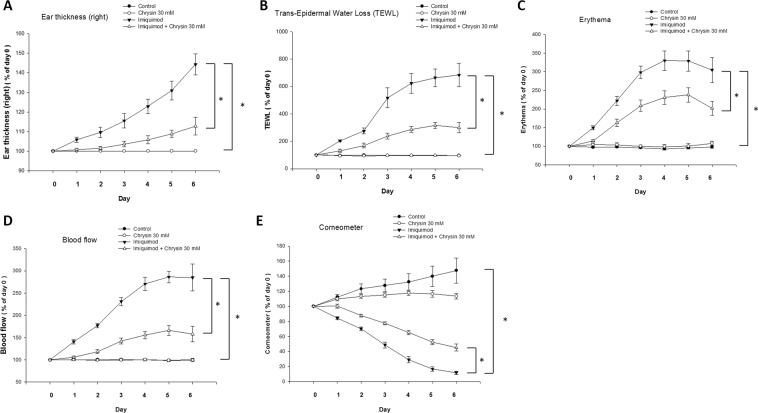
Chrysin improves physiology of mouse skin surface treated with IMQ. The effects of pre-treatment of chrysin on (**A**) ear thickness, and the physiological parameters such as (**B**) trans-epidermal water loss (TEWL), (**C**) erythema, (**D**) blood flow, and (**E**) skin hydration values of the skin surface of IMQ-treated mice were evaluated. The value of ear thickness and physiological parameters, including TEWL, erythema, and blood flow, were significantly lower compared to the IMQ only group (**A–D**). Skin hydration level was determined by Corneometer probe, and it increased after chrysin treatment following IMQ stimulation (**E**). Data represent the mean ± SEM from at least six independent experiments.

After IMQ stimulation, trans-epidermal water loss and values of physiological parameters, such as erythema, blood flow, and ear thickness, increased significantly. In addition, the surface skin hydration (corneometer) was greatly reduced. These results showed that IMQ induced an inflammatory state in the skin. The pretreatment by topical application of chrysin significantly reduced the trans-epidermal water loss, erythema, blood flow, and ear thickness, and increased the content of surface skin hydration. These findings indicated that chrysin plays an inhibitory role in IMQ-induced inflammation (Fig. 3).

### Chrysin inhibits signalling induced by TNF-α, IL-17A, or IL-22 in normal human epidermal keratinocytes (NHEKs)

**Figure 4:**
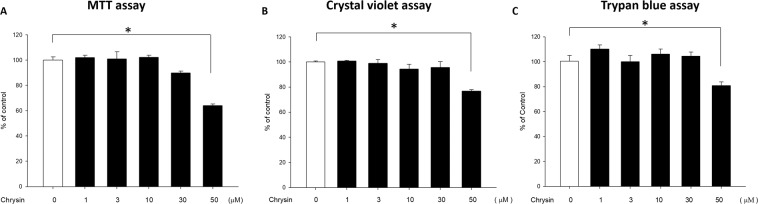
Chrysin exhibits no cytotoxicity in normal human epidermal keratinocytes (NHEKs). The (**A**) MTT assay, (**B**) crystal violet assay, and (**C**) trypan-blue exclusion method are the most common methods used to determine cell viability. We treated NHEKs with different concentrations of chrysin (1–50 μM), and the results showed that chrysin exhibited no cytotoxicity in the concentration range of 1–30 μM. However, at a concentration of 50 μM, the cell viability was decreased, it was shown that chrysin has cytotoxicity at this concentration. Therefore, in subsequent experiments, the concentrations of chrysin 3, 10, and 30 μM were selected. Results are expressed as a percentage of control value and are represented by mean ± SEM from at least three independent experiments. *p < 0.05 was considered to be statistically significant.

We first pretreated human primary keratinocytes with different concentrations of chrysin (0, 1, 3, 10, 30, and 50 μM), followed by MTT assay, crystal violet assay, and trypan blue assay. MTT assay revealed that pretreatment with different concentrations of chrysin did not cause cytotoxicity at concentrations between 0–30 μM; however, at 50 μM, chrysin began to cause toxicity in cells and affected cell viability (Fig. 4A). The results of crystal violet assay and trypan blue assay were consistent with that of MTT assay (Fig. 4B,C). As chrysin was not cytotoxic to NHEK at concentrations between 0–30 μM, we chose three concentrations of 3, 10, and 30 μM for further experiments.

**Figure 5:**
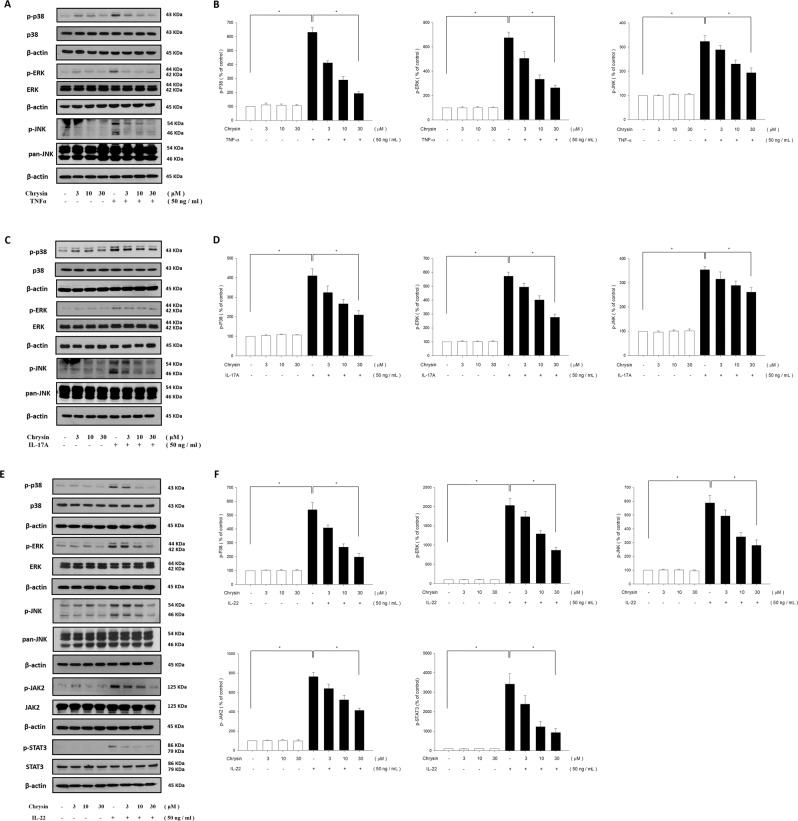
Chrysin inhibits phosphorylation of MAPK and JAK/STAT pathway components induced by TNF-α, IL-17A, and IL-22 in normal human epidermal keratinocytes (NHEK). NHEK were pretreated with different doses of chrysin, and stimulated with either (**A**) TNF-α, (**C**) IL-17A, or (**E**) IL-22, and the quantification data are shown in the right panel (**B**,**D**,**F**). The total protein was extracted from the cells and associated protein expression was determined via western blotting. The results are presented as a percentage of the control. *p < 0.05 was considered to be statistically significant.

TNF-α-stimulated NHEK cells showed a significant increase in levels of the mitogen-activated protein kinase (MAPK) pathway components, including p38 kinase, extracellular signal-regulated kinase (ERK), and c-Jun N-terminal kinase (JNK), when compared with those in the controls. Pretreatment with chrysin at various concentrations (3, 10, and 30 μM) significantly attenuated the TNF-α-stimulated increase in phosphorylation of p38 kinase, ERK, and JNK proteins (Fig. 5A,B). We also investigated the effect of chrysin on IL-17A-induced MAPK pathway activation using western blot analysis. The results showed that the phosphorylation of p38 kinase, ERK, and JNK activated only after IL-17A treatment. Pretreatment with chrysin at the working concentrations (3, 10, and 30 μM) inhibited the IL-17A-induced MAPK phosphorylation in a dose-dependent manner in NHEK (Fig. 5C,D). In addition, we examined the effects of chrysin on IL-22-induced phosphorylation of MAPK protein in NHEK using western blot analysis. The results showed that IL-22 treatment upregulated the phosphorylation of p38 kinase, ERK, and JNK, and chrysin pretreatment downregulated the activation of MAPK proteins following IL-22 stimulation (Fig. 5E,F).

Previous studies showed that the JAK-STAT pathway is a classical signal transduction pathway and is triggered by IL-22 stimulation. Therefore, we investigated the effects of chrysin on the activation of these related signalling molecules. After chrysin treatment, western blot was performed to identify the protein expression of phosphorylated JAK2 and STAT3. As shown in Fig. 5E,F, p-JAK2 and p-STAT3 levels were decreased by chrysin pretreatment compared with the IL-22 only group. These results suggested that TNF-α, IL-17A, and IL-22 indeed elicit psoriatic inflammation, and chrysin can significantly inhibit the production of these proinflammatory mediators.

### Chrysin suppressed TNF-α- or IL-17A-induced NF-κB pathway activation in NHEKs

**Figure 6:**
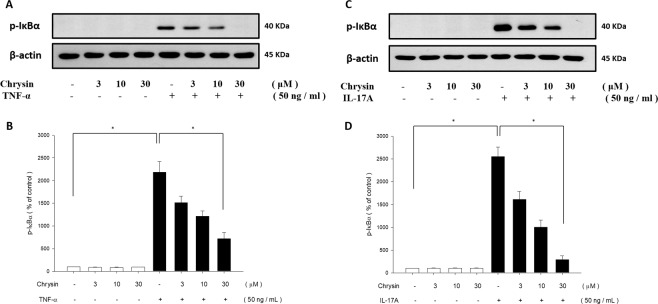
Chrysin inhibits TNF-α and IL-17A-induced phosphorylation of IKK/NF-κB pathway in NHEK. The phosphorylation of IκBα protein in both TNF-α (**A**) and IL-17A (**C**) only groups was significantly upregulated under pre-treatment with chrysin, and the expression of IκBα protein was inhibited by chrysin pre-treatment. The quantification data are shown in the below panel (B,D). The total protein was extracted from the cells and IκBα expression was determined via western blotting. The results are presented as a percentage of the control. *p < 0.05 was considered to be statistically significant.

NF-κB pathway is the key regulatory pathway during inflammation, and is considered to be a crucial mediator in the pathogenesis of psoriasis^42^. Therefore, we investigated the effects of chrysin on TNF-α- or IL-17A-mediated induction of α isoform of IκB (IκBα) in the NF-κB signalling pathway using western blot analysis. TNF-α- or IL-17A-stimulated NHEK cells showed a significant increase in IκBα protein levels when compared to the controls. In addition, pretreatment with chrysin downregulated the phosphorylation of IκBα protein. These results indicated that TNF-α and IL-17A treatments promote the NF-κB pathway, and this effect was reduced by chrysin pretreatment (Fig. 6A–D).

### Chrysin downregulated the mRNA expression of CCL20 and AMPs in NHEKs induced by TNF-α, IL-17A, or IL-22 recombination proteins

**Figure 7:**
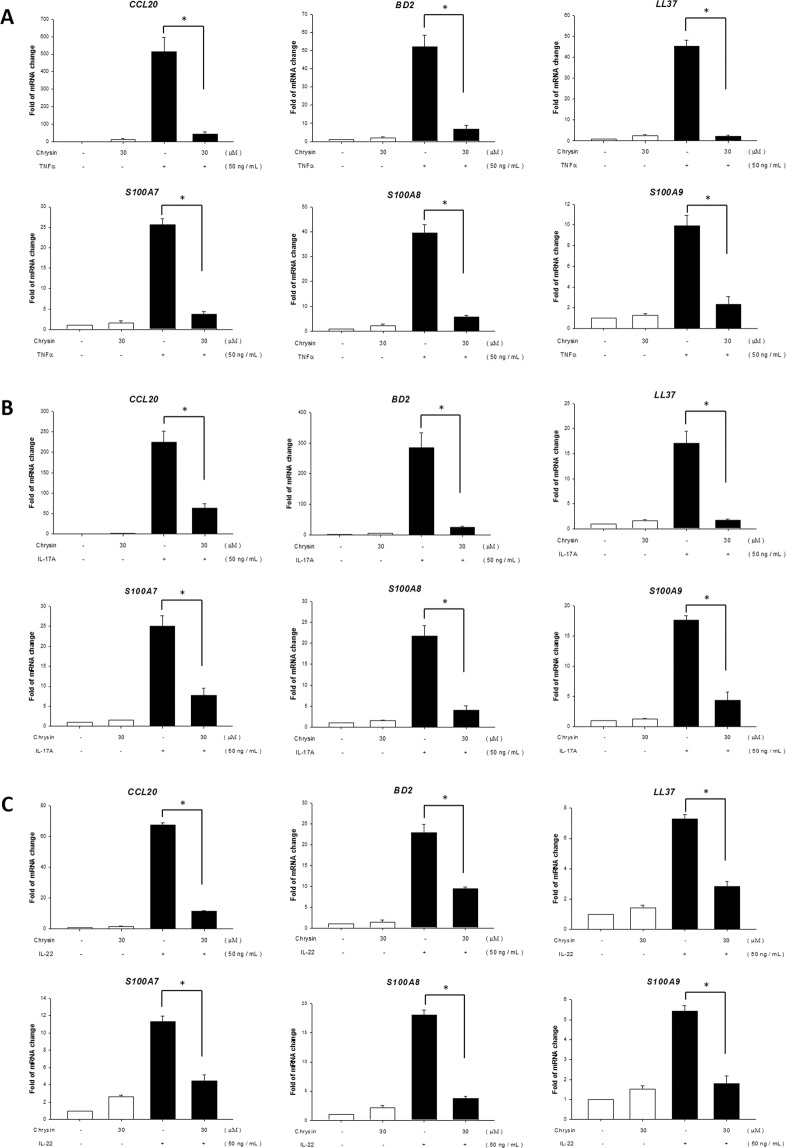
Chrysin downregulates the expression of TNF-α, IL-17A, and IL-22-induced genes at the mRNA level. After pre-treatment with chrysin, NHEK were stimulated with TNF-α (**A**), IL-17A (**B**), and IL-22 (**C**), and RT-qPCR was used to detect the effects of chrysin on the mRNA expression of CCL20, S100A7, S100A8, S100A9, hBD2, and LL-37. Data are expressed as fold induction of relevant mRNA sequences compared to untreated controls. Data represent mean ± SEM from at least three independent experiments performed in triplicates; *p < 0.05 was considered to be statistically significant.

In addition to providing a basic physical barrier, the skin is also an outpost of the immune response. These immune reactions are crucial responses to injury or infection, and can also regulate the skin symbiotic microbiota^43^. While the skin is infected with harmful pathogens (mostly microorganisms), keratinocytes are activated to secrete antimicrobial peptides and proteins (AMPs), cytokines, and chemokines to trigger an immune response. Chemokines, like CCL20, and AMPs, including cathelicidin, β-defensins, and S100 proteins, are speculated to intensify psoriasis lesions^44^, and excessive production of AMPs is an important characteristic of psoriasis lesions^43–45^. Previous studies have reported that TNFα, IL-17A, and IL-22 upregulate the expression of CCL20 and AMPs^46–48^. Therefore, in this study, we investigated whether chrysin can downregulate the expression of CCL20 and AMPs via TNFα, IL-17A, and IL-22 stimulation. Our results showed that the expression of CCL20, S100A7, S100A8, S100A9, hBD2, and LL-37 were significantly increased by TNFα, IL-17A, and IL-22 stimulation. In the group pretreated with chrysin, the expression of CCL20 and AMPs were significantly suppressed (Fig. 7A–C).

## Discussion

This study showed that chrysin can ameliorate the inflammation in psoriasis, and the underlying mechanism involves the regulation of three signalling pathways, namely MAPK, JAK-STAT, and IKK/NF-κB pathways. This is consistent with the results of our previous study, wherein chrysin was found to protect skin cells against the photodamage caused by UVA and UVB via the regulation of MAPK pathway and inhibition of ROS production^33^. In this study, we explored several factors that contributed to psoriasis, such as TNF-α, IL-17A, and IL-22 and found that chrysin could ameliorate the inflammatory reactions induced by these factors.

The most important pathological characteristics of psoriasis include hyperinflammation, epidermal keratinocyte proliferation and abnormal differentiation, and angiogenesis. The pathogenesis of psoriasis is a highly complex molecular mechanism, involving a variety of immune and inflammatory mediators. Several innate immune cells, adaptive immune cells, and epidermal keratinocytes are stimulated by various cytokines, which is the main cause for the sustained development of psoriatic lesions. However, based on previous research and clinical experiences, the immune and inflammatory cells release a variety of cytokines, especially TNF-α, IL-17A, and IL-22, which are believed to affect epidermal keratinocytes directly, and are the most important cytokines responsible for a variety of pathological processes.

Conventional treatments of psoriasis include topical and systemic medication^49,50^. Systemic steroids are widely used as anti-inflammatory drugs in the treatment of several skin diseases; however, they exhibit potential side effects, such as skin atrophy, striae, and telangiectasias^51^, or systemic response, including inhibition of the hypothalamic-pituitary-adrenal axis and other endocrinological complications, such as diabetes and osteoporosis^52^. In addition, the side effects of steroids in the cardiovascular, gastrointestinal, and neurological systems are well known. Besides these common side effects, some reports have indicated that psoriasis leads to a deterioration of the rebound phenomenon after systemic steroid withdrawal^53^. Moreover, morphological changes from non-pustular to pustular psoriasis can become difficult to control after discontinuation of steroid therapy, making it difficult to perform a risk-benefit analysis^54,55^. The use of steroids may also cause erythroderma in patients with psoriasis^56^. Although steroids are very effective as first-line treatments in many cases, the efficacy of the drug weakens over time in most cases, and higher doses are then required to maintain the treatment response. Therefore, steroids are not conducive to long-term use^57^. In contrast biological agents, which are proteins produced by organisms, have advantages, such as lower side effects, less hepatotoxicity and nephrotoxicity, and selective towards immune system. However, their disadvantages are that they are expensive and less popular. At present, the main biological agents are antibodies and agents against TNF, IL-12/23, IL-17, JAK, and IκB kinase^58^. TNF therapy has long been used to treat various inflammatory diseases, including psoriasis^59^, and mainly neutralizes TNF secretion by inflammatory cells. IL-12/23 Inhibitors interfere with IL-12 and IL-23-mediated cell signal transduction^60^, while IL-17 inhibitor neutralizes the effects of IL-17A^61^ and the downstream signalling pathways. In psoriasis, JAK inhibitors show good therapeutic effects. In a mouse model of contact dermatitis, topical application of JAK inhibitors could effectively inhibit the infiltration of lymphocyte, the phosphorylation of STAT3, and the proliferation of keratinocyte^62^. Recently, many studies have speculated that NF-κB pathway may be a new target for the treatment of psoriasis, and IκB kinase inhibitor can improve the severity of psoriasis^63^. However, compared with steroids and biological agents, Chinese herbal medicine ingredients are more advantageous, as they can be isolated from natural plants, can be conveniently obtained, have no usage restrictions, and show only few or no side effects. In modern medicine, there are more and more Chinese medicine treatments for psoriasis are extensively investigated^64^. Indigo naturalis has been used as a traditional Chinese medicine for the treatment of skin diseases. In recent years, many reports indicated that topical application of indigo naturalis and indirubin were effective and safe for the treatment of psoriasis, especially plaque psoriasis and nail psoriasis, and it also has the inhibitory effect on the inflammatory response of human neutrophils. It has also been found that indigo naturalis up-regulated the expression of claudin-1 and the function of tight junction in human epidermal cells^65–67^. However, although Chinese herbal medicine can be an alternative, safety, adverse effects and efficacy of long-term medication are still important issues that need to be addressed in the future. It is important to identify novel herbal ingredients that can be efficiently and safety used in abovementioned therapeutic approaches/strategies against psoriasis and other skin inflammatory diseases.

In this study, the topical application of chrysin suppressed the IMQ-induced psoriasis-like skin inflammation in the murine model, ameliorated the skin-related physiology, including trans-epidermal water loss (TEWL), erythema, blood flow speed, and ear thickness, and increased surface skin hydration (corneometer). Furthermore, pretreatment with chrysin reduced the IMQ-induced inflammation and infiltration of inflammatory cells, as observed in the histopathological analysis. We also found that chrysin effectively suppressed the downstream responses of these cytokines in epidermal keratinocytes, including phosphorylation of the MAPK pathway, activation of IκBα in the NF-κB pathway, and triggering of the JAK-STAT pathway at protein levels. Furthermore, chrysin treatment downregulated the expression of CCL20 and antimicrobial peptides (AMPs), consisting of S100A7, S100A8, S100A9, hBD2, and LL-37, at mRNA levels.

Many skin diseases, such as atopic dermatitis, contact dermatitis, psoriasis, and even ultraviolet radiation are related to skin inflammation. Inflammation of the skin is often accompanied by an increase in vascular permeability, together with the release of NO and prostaglandins, which trigger accumulation of tissue fluid in the lesion and occurrence of oedema. At the same time, leukocytes (especially neutrophils) also migrate to the skin lesions. The production of matrix metalloproteinase, in particular MMP-9, degrades the main structural component of connective tissue, collagen, in the epidermis and dermis^68^, which contributes to such migration of leukocytes^69^. Other cytokines released from epidermal cells or antigen-specific cells play a crucial role in inflammation of the skin, such as TNF-α, IL-1, and IL-6. Therefore, the above mentioned mediators can be used as indicators for exploring skin inflammation^4^. TNF-α plays a key role in inflammatory and barrier-impaired skin diseases, and also affects the regulation of many downstream molecules, such as cytokines, chemokines, enzymes, and proteins. Therefore, by elucidating the effects of Chinese herbal medicines on the cellular signalling pathways induced by TNF-α, we could reduce the effects of these inflammatory molecules, and identify the potential and feasibility of Chinese herbal medicines for the treatment of inflammatory skin diseases.

Cytokines involved in both Th1 and Th17 pathways are found in the skin of psoriasis patients, including IL-17A, IL-17F, IL-19, IL-20, IL-22, IL-23, IL-24, IL-26, and TNF-α, which can be detected in serum and lesions. The synergistic effect of IL-17 and IL-22 promotes the expression of antimicrobial peptides in keratinocytes, such as β-defensin-2 (BD-2), S100A7 (psoriasin), cathelicidin (LL37), and S100A8/9 (calprotectin), all of which may lead to the development of psoriasis in individuals with a higher resistance to skin infections^70,71^. In this study, the results showed that three cytokines, TNF-α, IL-17A, and IL-22, induced mRNA expression of CCL20 and antimicrobial peptides, and chrysin pretreatment downregulated their mRNA levels significantly. In recent years, an increasing number of studies have reported that interleukin-36 (IL-36), which belongs to the IL-1 superfamily, affects the balance between pro-inflammatory and anti-inflammatory branches, and easily leads to tissue inflammation. IL-36 cytokines are mainly expressed at the barrier sites in the body, such as the skin epithelium, and the spectrum of psoriasis is one of the famous examples^72^. Therefore, IL-36 is likely to be an important diagnostic tool for dermatitis, and additional keratinocyte-linked cytokines like the inflammasome-related IL-1β, IL-36α, and IL-36γ should be investigated in the future. Previous studies have indicated that IL-17 exhibits the ability to activate transcription factors, such as NF-κB, in many cell types, including fibroblasts, macrophages, chondrocytes, intestinal epithelial cells, and myofibroblasts of the colon and pancreas^73^. When cells are stimulated by IL-17, the IL-17 receptor (IL-17R) is triggered to activate ERK1 and ERK2 and the stress-induced JNK-1 and JNK-2, as well as the p38 MAPK pathway; these signal transduction pathways contribute to the upregulation of IL-6, IL-1, and NF-κB^74^. Our results showed that the addition of TNF-α, IL-17A, and IL-22 induced the phosphorylation of p38, ERK, and JNK, and TNF-α and IL-17A activated the expression of IκBα in the NF-κB pathway. Previous studies have reported that IL-22 induces the phosphorylation of IKK and IκB in HaCaT cells^75^. However, we have not explored the effects of IL-22 on the NF-κB pathway in this study. If we can further explore the effects of this pathway, we can better understand the mechanism of chrysin on the pathogenesis of psoriasis. The phosphorylation of these related proteins was suppressed by the pretreatment of chrysin in a dose-dependent manner. These results demonstrated that chrysin effectively improved psoriasis-associated inflammation and exhibited anti-psoriatic potential.

With the advent of cellular molecular biotechnology, it has become increasingly clear that many molecules related to the skin barrier, as well as molecules that maintain skin hydration, such as filaggrin and junction protein, play a decisive role in maintaining normal physiological functions of the skin. Therefore, exploring and reducing the external factors that affect the above skin properties is an important research area in pathology and future drug development. Cornification (or keratinization) is the process of terminal differentiation of epidermal keratinocytes and plays an important role in the formation of the skin barrier^76,77^. In psoriatic lesions, abnormal differentiation of keratinocytes is observed. In particular, the expression of some crucial differentiation markers, such as keratin 10, loricrin, and filaggrin, is suppressed, leading to hypogranulosis in the psoriatic lesion epithelium. The three cytokines, TNF-α, IL-17A, and IL-22, affect the differentiation of keratinocytes and result in downregulation of the abovementioned differentiated proteins. In addition, proliferation and thickening of the skin in psoriatic lesions can also be observed, which is related to the hyperplasia of keratinocytes. Previous evidence showed that among the several cytokines that are pathogenic to psoriasis, IL-22 most likely promotes the proliferation of keratinocytes^78,79^. In the inflammatory pathway, we found that chrysin can effectively inhibit the expression of inflammation-related proteins and mRNAs induced by TNF-α, IL-17A, and IL-22. In future, we hope to address whether chrysin can reverse the inhibition of TNF-α, IL-17A, or IL-22-induced differentiation in keratinocytes in psoriasis.

In conclusion, the current study demonstrates the potential of chrysin in the treatment of inflammatory skin diseases, such as psoriasis, via its ability to relieve their symptoms. The findings also suggest that it may be useful as a daily health care supplement for prevention of inflammation.

## Materials and Methods

### Ethics statement

All animal experiments in this study were approved by the Institutional Animal Care and Use Committee of Fu Jen Catholic University (approval #A10367). The principles of the 3Rs (Replacement, Reduction, and Refinement) were followed to optimize the experimental design. The human primary epidermal keratinocytes were cells from human foreskins. The foreskins were provided by the Mackay Memorial Hospital, after obtaining the consent for use (#13MMHIS022) from the Institutional Review Board. There was no interaction between the researcher and the foreskin donors, and the foreskins do not have any relevant information to identify the donors. All experiments were performed in accordance with relevant guidelines and regulations.

### Materials

The chrysin used in this study was purchased from Sigma-Aldrich (St Louis, MO; CAS: 480-40-0) with a purity of 97% and was dissolved in dimethyl sulfoxide (DMSO). Antibodies (Ab) against p-ERK and ERK were purchased from Santa Cruz Biotechnology (Santa Cruz, CA, USA). Abs against p-IκBα, p-STAT3, P38, STAT3, p-JAK2, JAK2, and β-actin were purchased from Cell Signaling Technology, Inc. (Beverly, MA, USA). Abs against p-P38, p-JNK and JNK were purchased from R&D Systems (Minneapolis, MN). TNF-α, IL-17A, and IL-22 were purchased from PeproTech (Rocky Hill, NJ).

### Animals

Male mice BALB/c (8–11 weeks) were used for all experiments. Mice were purchased from the National Laboratory Animal Center, Taipei, Taiwan. Animals were individually housed in polypropylene cages with controlled temperature (21–25 °C), light (12/12 h light/dark cycle), and humidity (60 ± 5%). All animals had *ad libitum* access to standard food and water.

### IMQ-induced psoriasis-like skin inflammation in mice

Chrysin (30 mM) or vehicle was applied to the dorsal shaved back and the right ears of mice. After 1 h, mice received a 62.5 mg topical dose of commercially available imiquimod cream (Aldara 5%; Meda AB, Solna, Sweden) or vehicle cream (Vaselina Pura, Laboratorios Rida, Valencia, Spain) on the same positions for six consecutive days. Skin physiology-related values, including trans-epidermal water loss (TEWL), erythema, skin hydration by MPA-580 (Courage & Khazaka, Cologne, Germany), and blood flow were measured with FLO-N1 (Omegawave, Tokyo, Japan) daily before chrysin treatment. The Mexameter® MX 18 is available as a probe that connects to the MPA systems, which is a tool to measure the two components, mainly responsible for the colour of the skin: melanin and haemoglobin (erythema) by reflectance. For use, the probe was pressed on the measurement site for ~1 s to measure the melanin and erythema indices. FLO-N1, a non-contact type of instrument, was used to measure tissue blood flow, blood volume, and flow velocity. In addition, the thickness of both ears in mice were measured and photographed for indicating the changes in the appearance of the skin and ears. At the end of the experiment, mice were sacrificed and tissues were collected and stored at −80 °C for subsequent homogenization or fixation in formalin.

### Histopathological analysis

Mouse tissues were fixed with 4% paraformaldehyde at 4 °C overnight. Routine methods were used to prepare formalin-fixed paraffin-embedded blocks of the mice’s skin tissues. These tissues were then cut into 5-μm sections, and stained with haematoxylin and eosin (H&E). ZEISS Axioskop 40 Inverted System microscope (NY, United States) and SPOT Cam software (Sterling Heights, MI) were used to visualize the Images from H&E staining. Psoriasis Area and Severity Index (PASI) is an objective scoring system. The erythema, scaling, and thickness were scored independently from 0 to 4 (0 no infection,1 mild, 2 intermediate, 3 severe, 4 very severe), and the total score was used as an index of psoriasis severity (scores 0–12).

### Cell culture

The primary keratinocytes were isolated from human foreskin tissue, and were grown in Keratinocyte-SFM (Gibco BRL/Invitrogen, Carlsbad, CA). The primary keratinocytes were used between passages 2 to 4 in this study. Normal human keratinocytes were plated in 35-mm culture dishes, and 24 h prior the stimulus, keratinocyte medium was switched and chrysin (3, 10, or 30 μM) was added. The control medium contained an equal volume of DMSO. Finally, cells were stimulated with either TNF-α (50 ng ml^−1^), IL-17A (50 ng ml^−1^), or IL-22 (50 ng ml^−1^) from PeproTech (Rocky Hill, NJ).

### Cell viability assays (MTT, trypan blue assay, and crystal violet assay)

Cell viability was determined as previously described^32,34^ by MTT, trypan blue, and crystal violet assays. In MTT assay, cells were pretreated with DMSO or chrysin for 24 h. After a brief wash, MTT (0.5 mg/mL in Keratinocyte-SFM) was used for the quantification of metabolically-active live cells and were analysed photometrically at 550 nm. In crystal violet assay, cells were treated as described, fixed with methanol, and then stained with 0.1% crystal violet solution for 1 h staining. Then, the cells were washed thrice with double-distilled water, followed by acetic acid to dissolve the cells. The optical density (OD) was then read using a Tecan Sunrise spectrophotometer (Tecan, Crailsheim, Germany) at 550 nm. The Trypan Blue exclusion method was performed according to the manufacturer’s protocols. The normal human keratinocytes were pretreated with chrysin, and then suspended and stained with equal volume of trypan blue dye. The cells were counted using a dual-chamber haemocytometer and a light microscope.

### Western blot analysis

After 5 and 15 min of stimulation using TNF-α, IL-17A, or IL-22, the total proteins were extracted by applying RIPA lysis buffer. Protein concentration was measured with the Pierce protein assay kit (Pierce, Rockford, IL). The proteins were separated by electrophoresis on 10% SDS–polyacrylamide gels, and then transferred onto a PVDF membrane (Millipore, Darmstadt, Hesse, Germany). After blocking with 5% non-fat dry milk for 1 h at room temperature, the membranes were probed using the indicated specific antibodies and visualized with ECL solution.

### Real-time quantitative RT-PCR

After 6 and 8 h of stimulation with TNF-α, IL-17A, or IL-22, total RNA was isolated using the total RNA isolation kit (GeneDireX®, Vegas, NV) according to manufacturer’s instructions and reverse-transcribed into cDNA using SuperScript™ III First-Strand Synthesis System kit (Invitrogen, Carlsbad, CA). The qPCR was performed using the CFX96™ Real-Time PCR Detection System (Bio-Rad, Hercules, CA), following the previously described conditions^34^ with SYBR green (Kapa Biosystems, Wilmington, MA). Primer sequences used in the PCR reactions are listed in Table 1. Data were normalized relative to β-actin expression and evaluated using the equation: fold change = 2^−ΔΔCT^.

**Table 1:** Primer sequences for RT-qPCR.

Gene	Primer sequence (5′-3′)
Human CCL20^80^	F: TACTCCACCTCTGCGGCGAATCAGAA
	R: GTGAAACCTCCAACCCCAGCAAGGTT
Human S100A7^81^	F: GCATGATCGACATGTTTCACAAATACAC
	R: TGGTAGTCTGTGGCTATGTCTCCC
Human S100A8^82^	F: TGAAGAAATTGCTAGAGAC
	R: CTTTATCACCAGAATGAGGA
Human S100A9^78^	F: GCTCCTCGGCTTTGACAGAGTGCAAG
	R: GCATTTGTGTCCAGGTCCTCCATGATGTGT
Human BD2^83^	F: CCAGCCATCAGCCATGAGGGT
	R: GGAGCCCTTTCTGAATCCGCA
Human LL-37^84^	F: GCAGTCACCAGAGGATTGTGAC
	R: CACCGCTTCACCAGCCC
Human β-Actxin^85,86^	F: CGGGGACCTGACTGACTACC
	R: AGGAAGGCTGGAAGAGTGC

### Statistical analysis

Data were expressed as the mean ± SEM of *in vivo* experiments at least six mice per treat group, and three cell culture replicates in the *in vitro* experiments by using GraphPad Prism Program 6 software (GraphPad Software San Diego, CA). Comparison of the mean difference with and without chrysin treatment was made using one-way ANOVA followed by Dunnett’s t-test for multiple comparisons. We considered *p* < 0.05 to be statistically significant.
